# Who came to the rescue? Sources of informal support to older Europeans before, during and after the COVID-19 pandemic

**DOI:** 10.1093/ageing/afaf034

**Published:** 2025-02-21

**Authors:** Judite Gonçalves, France Weaver

**Affiliations:** Imperial College London School of Public Health, White City Campus, 90 Wood Lane, London W12 0BZ, UK; NOVA University Lisbon NOVA School of Business and Economics, Campus de Carcavelos, Rua da Holanda, 12775-405 Carcavelos, Portugal; NOVA National School of Public Health, Public Health Research Centre, Comprehensive Health Research Center, CHRC, NOVA University Lisbon, Avenida Padre Cruz, 1600-560 Lisboa, Portugal; Department of Health Policy, Management, and Leadership, West Virginia University School of Public Health, 64 Medical Circle Drive, Morgantown, WV 26505, USA

**Keywords:** COVID-19, pandemic, informal care, activities of daily living, Survey of Health, Ageing and Retirement in Europe (SHARE), older people

## Abstract

**Background/Objective:**

The COVID-19 pandemic disrupted the provision of informal care in major ways. This study documents the prevalences of informal support with (instrumental) activities of daily living (IADL and ADL) before, during and after the pandemic, distinguishing between children, other relatives and friends/neighbours and focusing on individuals 50 years and older across 27 European countries.

**Methods:**

This longitudinal analysis relies on the Survey of Health, Ageing and Retirement in Europe (SHARE)’s Wave 8 (2019), two Corona surveys (2020 and 2021) and Wave 9 (2022). Linear probability models adjusted for individual fixed effects and time-varying confounders were used to estimate prevalences of informal support over time.

**Results:**

During the pandemic, the prevalences of informal support with both IADL and ADL from all three groups of caregivers increased significantly (*P* < 0.01), to return to their pre-pandemic levels by 2022. For example, the adjusted likelihood of IADL help from children increased from 18.5% (2019) to 36.6% (2020) and 42.5% (2021), then dropped back to 19.7% in 2022. Friends and neighbours played a critical role, with the adjusted likelihood of IADL help going from 8.8% (2019) to 29.7% (2020), then down to 18% (2021) and 8.9% (2022).

**Conclusions:**

Future emergency and disaster preparedness plans should contemplate the various sources of informal care, including support measures to non-relative caregivers, as those helpers may be able to rapidly respond to unexpected crisis.

## Key Points

- We document trajectories in informal support to European older adults around the COVID-19 pandemic.
- We distinguish between support from out-of-household children, other relatives and friends/neighbours.
- Compared to 2019, informal support from all sources increased substantially in 2020 and 2021.
- Children especially stood out as sources of support during 2020–21, and so did friends and neighbours.
- In 2022, informal support was back to pre-COVID-19 prevalences.

## Introduction

Around 12% of European adults 50 years and older (50+) have long-term care (LTC) needs, and this share will continue to rise [1]. Informal carers provide most of LTC [2, 3]. Across 10 European countries and the US, 54% of 65+ individuals who receive LTC rely solely on informal care and 21% receive a combination of formal home care and informal care [2]. Spouses and (female) children provide the bulk of informal care hours [2, 4, 5]. Increasing divorce rates, decreasing numbers of children and other demographic changes cast doubt on the ability of spouses and children to meet the LTC needs of ageing populations in the future [6].

Studies show that relatives other than spouses and children as well as friends and neighbours are also significant informal caregivers [5, 7–11]. Those sources provided 30% of informal care hours to 65+ care recipients—especially those single and less able to rely on children—across nine Western European countries in 2004–07 [8]. Understanding better the contributions of those other groups of caregivers is needed, to propose both policies that leverage their potential to meet growing and changing LTC needs and policies that support all types of caregivers, regardless of family ties [12–14].

Unanticipated events such as the COVID-19 pandemic create natural laboratories to study how different sources of informal care respond to support older adults in times of crisis. The pandemic and the restrictions introduced to control the spread of the disease prompted unexpected changes to the mix of formal and informal LTC to older adults. Stay-at-home orders, travel restrictions, physical distancing and fear of infection limited the mobility of older adults and severely disrupted existing LTC arrangements, including access to formal home care and nursing home care [15–17]. Worsening physical and mental health and lower opportunity cost of providing informal care may also have induced changes [18, 19]. The complex interplay between LTC needs and available support is amplified by substitutions and complementarities between different sources of formal and informal care [20]. For example, individuals receiving care from multiple sources in normal times may have concentrated all help into one single source to minimise risk of contagion [21]. Several studies documented increases in informal support provided to/received by older adults during the initial stages of the pandemic [15–17, 22–25], with one exception from Austria [26]. Those increases occurred across the board, specifically from outside the household and from a range of sources, including friends and neighbours [23, 27, 28]. Literature on less sudden events, such as economic crises, has also shown increases in informal support, especially from non-cohabiting caregivers, such as friends and non-co-residing family members [18].

Previous studies on informal support during the pandemic that used the same data source as in the present study were limited to the first few months of the pandemic and based on one cross-sectional snapshot [15, 16], with one study looking at 2020 and 2021 [24]. Most of the other studies are single-country or focused on specific populations, such as persons with dementia [17, 23, 25–31].

This study takes advantage of the natural social experiment that was the COVID-19 pandemic to investigate how various sources of informal care from outside the household ‘came to the rescue’ in that time of need. It estimates adjusted prevalences of informal care provided by children, other relatives and friends/neighbours prior (2019), during (2020 and 2021) and after the COVID-19 pandemic (2022). We make two main contributions to current evidence. First, we document the trends in the prevalence of support across helpers over time, for the same individuals and adjusting for changing health and ability to make ends meet (i.e. longitudinal study). Second, our sample period starts before the onset of the pandemic and continues until after the main phases of the pandemic ended and restrictions were lifted. Our results inform on whether some sources of support were unlocked that could help meet future LTC needs or whether helpers provided mostly transitory support.

## Methods

### Data and sample

The Survey of Health, Ageing and Retirement in Europe (SHARE) is a multidisciplinary, cross-national panel survey that includes nationally representative samples of individuals 50+ and their partners irrespective of age, in 28 European countries and Israel. We used the Regular Wave 8 (interviews between October 2019 and March 2020, i.e. before the COVID-19 outbreak), the two Corona Waves (June–September 2020 and June–August 2021), and the Regular Wave 9 (October 2021–October 2022, i.e. after the main phases of the pandemic—a timeline of data collection, recall periods and the pandemic is available in Appendix 1 in the Supplementary Data file).

To focus on individuals who may need informal support, the sample was restricted to individuals with any limitation in the activities of daily living (ADL)―e.g. dressing, bathing―or instrumental activities of daily living (IADL)―e.g. cooking, shopping― at baseline. This resulted in ⁓9600 individuals with at least one ADL or IADL limitation in Wave 8, and a total of ⁓26 300 person-wave observations (unbalanced panel—the sample split by country and wave is available in Appendix 2 in the Supplementary Data file).

### Informal help

Our focus was informal care received from three sources outside the household: children, other relatives and friends/neighbours. We distinguished between help received for ADL or IADL limitations. As a result, there are six outcome variables. IADL help outcomes are available in all four waves, whereas ADL help outcomes are not available in the first Corona wave. The exact survey questions in the different waves are presented in Appendix 3 in the Supplementary Data file.

### Key variable of interest: time

Time is captured by three binary indicators for 2020 (first Corona wave), 2021 (second Corona wave) and 2022 (Regular Wave 9) for IADL help, and 2021 and 2022 for ADL help, with 2019 serving as the reference year. These indicators capture the total effect of the COVID-19 pandemic and other time trends.

### Other variables

Individual fixed effects adjusted for time-invariant characteristics—including gender, country of residence, personality traits, socioeconomic status, family composition and availability of help (e.g. number of children)—to the extent that these variables remained constant over the sample period, which is likely for most individuals. We further adjusted for economic wellbeing and health status; two factors more likely to change over time, in particular because of the COVID-19 pandemic. Economic wellbeing was measured by the ability to make ends meet: with great difficulty (reference), some difficulty, fairly easily, or easily. Physical and mental health were captured by a set of binary indicators: self-assessed health―excellent, very good, good, fair, poor (reference); prescription drugs for high cholesterol, high blood pressure, coronary diseases, other heart diseases, diabetes and chronic bronchitis; frailty symptoms—any falls, fear of falling, dizziness and fatigue; feeling sad or depressed, sleeping difficulties and loneliness―never, sometimes, often (reference).

### Statistical analyses

The analyses were conducted in Stata 18. For each of the six outcomes, we estimated five linear fixed effects models. Models 1 and 2 were equivalent, except Model 1 was unweighted. Models 2–5 incorporated survey weights to obtain results representative of the European population aged 50+ in 2019. Model 2 included the covariates described in the previous section and is our preferred specification. We wanted to capture the full effects of the pandemic, disentangled from the natural ageing process captured by the health indicators. Model 3 included difficulties accessing home care as a covariate. Access to formal home care was particularly challenging in the first phase of the pandemic, which likely altered recourse to informal care. Yet, this can be seen as an effect of the pandemic and we preferred to let the time indicators capture that, omitting this covariate in our preferred Model 2. Compared to Model 2, Model 4 excluded all health-related covariates. Lastly, Model 5 is equivalent to Model 2, but estimated on the balanced panel. As data were longitudinal, standard errors were adjusted for heteroskedasticity and clustering of observations from the same individual over time.

Model 2 was used to predict the mean likelihoods of receiving help in 2019, 2020, 2021 and 2022, at the means of the explanatory variables. Those predictions allowed us to plot the adjusted trajectories of the likelihoods of receiving IADL and ADL support and compare the relative importance of different helpers over time.

Lastly, we re-estimated Model 2 and computed the respective predictions separately for females living alone, males living alone and individuals residing with someone else, as living arrangement and gender are known to be associated with need for and acceptance of informal support [32]. Lastly, we re-estimated Model 2 separately by country because of their different institutional and cultural characteristics associated with informal care, as well as their various policy decisions regarding COVID-19 [2, 14].

## Results

### Sample characteristics

Our sample of 9645 individuals with any IADL or ADL limitation in 2019 included a total of 26 228 observations over the four waves. In 2019, the unadjusted proportions of individuals receiving IADL help from children, other relatives and friends or neighbours were 18.9%, 9.4% and 9.8%, and the proportions of individuals receiving ADL help from each of the three sources were 5.6%, 2.8% and 1%, respectively (Table 1). These proportions increased substantially in 2020 and even further in 2021. In 2022, when the pandemic had faded, the proportions of individuals receiving informal help were comparable to those observed at baseline. As expected, across all 4 years and for both IADL and ADL support, children were the main providers of informal support from outside the household. Refer to Table 1 for descriptions of the covariates and Appendix 4 in the Supplementary Data file for additional sample characteristics.

**Table 1:** Descriptive statistics by year (proportions, weighted to reflect countries’ 50+ populations in 2019).

	2019	2020	2021	2022
Help with IADL from				
Children	0.189	0.370	0.422	0.188
Other relatives	0.094	0.237	0.128	0.082
Friends/neighbours	0.098	0.282	0.170	0.093
Help with ADL from				
Children	0.056	n.a.	0.089	0.048
Other relatives	0.028	n.a.	0.029	0.014
Friends/neighbours	0.010	n.a.	0.028	0.006
Ability to make ends meet				
With great difficulty	0.186	0.120	0.109	0.156
With some difficulty	0.282	0.286	0.285	0.280
Fairly easily	0.290	0.339	0.338	0.312
Easily	0.242	0.255	0.269	0.252
Self-assessed health				
Poor	0.308	0.212	0.223	0.271
Fair	0.427	0.372	0.453	0.407
Good	0.214	0.336	0.264	0.260
Very good	0.037	0.060	0.051	0.049
Excellent	0.013	0.019	0.009	0.013
Drugs for:				
High cholesterol	0.303	0.345	0.349	0.330
High blood pressure	0.588	0.638	0.639	0.611
Coronary diseases	0.177	0.267	0.214	0.182
Other heart diseases	0.198	0.244	0.250	0.200
Diabetes	0.196	0.208	0.216	0.203
Chronic bronchitis	0.064	0.092	0.064	0.050
Frailty symptoms				
Falls	0.218	0.185	0.209	0.209
Fear of falling	0.366	0.390	0.450	0.382
Dizziness	0.327	0.283	0.329	0.309
Fatigue	0.465	0.446	0.534	0.448
Sad or depressed	0.620	0.430	0.482	0.597
Sleeping troubles	0.542	0.415	0.450	0.502
Feels lonely:				
Often	0.178	0.160	0.164	0.168
Sometimes	0.299	0.313	0.329	0.295
Never	0.523	0.527	0.506	0.537
Difficulties accessing home care	0.000	0.039	0.013	0.000
# observations	9645	5836	4863	5884

Note: These sample sizes exclude observations with missing information on any of the covariates considered, but not on the outcome variables to maximise all available observations.

### Model results

In line with the unadjusted proportions in Table 1, the coefficients of the 2020 and 2021 time indicators were positive and statistically significant (*P* < 0.01), with the exception of ADL help from other relatives (Table 2). For example, the likelihood of receiving IADL help from children was 18 percentage points (pp) higher in 2020 compared to 2019, and 24 pp higher in 2021 compared to 2019. For all outcomes, there were no statistically significant differences between the likelihoods of receiving informal support in 2019 and 2022. Results from alternative specifications are highly comparable and confer robustness to these results (see Appendices 5–10 in the Supplementary Data file).

**Table 2:** Associations with the likelihoods of informal support from various sources, full results from the preferred model (Model 2)

	IADL help from			ADL help from		
	Children	Other relatives	Friends/neighbours	Children	Other relatives	Friends/neighbours
Year (ref.: 2019)						
2020	0.180^*^^*^^*^	0.153^*^^*^^*^	0.209^*^^*^^*^			
	(0.012)	(0.011)	(0.011)			
2021	0.240^*^^*^^*^	0.045^*^^*^^*^	0.092^*^^*^^*^	0.045^*^^*^^*^	0.008	0.023^*^^*^^*^
	(0.012)	(0.009)	(0.010)	(0.007)	(0.005)	(0.004)
2022	0.011	−0.001	0.001	0.003	−0.005	−0.001
	(0.009)	(0.008)	(0.008)	(0.006)	(0.003)	(0.002)
Ability to make ends meet (ref.: with great difficulty)						
With some difficulty	0.017	0.011	0.014	−0.007	−0.016	0.008
	(0.019)	(0.016)	(0.016)	(0.018)	(0.014)	(0.007)
Fairly easily	0.035	0.043^*^^*^	0.026	−0.004	−0.008	0.015^*^^*^
	(0.022)	(0.019)	(0.019)	(0.018)	(0.012)	(0.008)
Easily	0.024	0.053^*^^*^	0.020	−0.014	−0.007	0.021^*^^*^
	(0.024)	(0.021)	(0.022)	(0.019)	(0.015)	(0.009)
Self-assessed health (ref.: poor)						
Fair	−0.010	−0.019	−0.028^*^^*^	−0.015	−0.025^*^^*^^*^	−0.010
	(0.016)	(0.013)	(0.013)	(0.012)	(0.009)	(0.007)
Good	−0.023	−0.037^*^^*^	−0.053^*^^*^^*^	−0.013	−0.033^*^^*^^*^	−0.013^*^
	(0.020)	(0.016)	(0.017)	(0.013)	(0.010)	(0.007)
Very good	−0.043	−0.054^*^^*^	−0.100^*^^*^^*^	−0.034^*^^*^	−0.014	−0.011
	(0.026)	(0.023)	(0.025)	(0.017)	(0.023)	(0.014)
Excellent	0.017	−0.070^*^	−0.092^*^^*^	−0.014	−0.020	−0.009
	(0.039)	(0.039)	(0.047)	(0.019)	(0.013)	(0.010)
Drugs for:						
High cholesterol	−0.018	−0.027^*^^*^	−0.038^*^^*^	−0.030^*^^*^	−0.008	−0.009
	(0.015)	(0.013)	(0.015)	(0.015)	(0.006)	(0.006)
High blood pressure	−0.001	0.002	0.004	0.005	−0.009	−0.012^*^^*^
	(0.016)	(0.013)	(0.014)	(0.009)	(0.009)	(0.006)
Coronary diseases	0.023	−0.012	0.018	0.015	−0.017^*^^*^	−0.003
	(0.016)	(0.015)	(0.014)	(0.012)	(0.007)	(0.006)
Other heart diseases	−0.004	−0.007	0.001	0.001	−0.001	−0.001
	(0.015)	(0.013)	(0.012)	(0.011)	(0.008)	(0.007)
Diabetes	−0.024	0.020	−0.011	0.010	0.015	0.004
	(0.023)	(0.023)	(0.024)	(0.018)	(0.012)	(0.011)
Chronic bronchitis	0.001	0.052^*^^*^	0.013	−0.011	0.017	0.004
	(0.025)	(0.025)	(0.024)	(0.016)	(0.016)	(0.014)
Frailty symptoms						
Falls	0.005	0.007	0.011	0.014	0.018^*^^*^	0.001
	(0.016)	(0.014)	(0.013)	(0.011)	(0.009)	(0.005)
Fear of falling	0.010	0.012	0.011	0.011	−0.013^*^	0.002
	(0.014)	(0.012)	(0.012)	(0.011)	(0.007)	(0.005)
Dizziness	0.031^*^^*^	0.020^*^	0.013	0.013	0.011^*^	0.004
	(0.013)	(0.011)	(0.011)	(0.009)	(0.006)	(0.005)
Fatigue	0.004	−0.003	−0.017	−0.001	0.006	−0.003
	(0.013)	(0.011)	(0.011)	(0.010)	(0.007)	(0.004)
Sad or depressed	0.044^*^^*^^*^	0.020^*^^*^	0.035^*^^*^^*^	0.022^*^^*^	0.005	0.004
	(0.012)	(0.010)	(0.011)	(0.009)	(0.005)	(0.005)
Sleeping troubles	−0.009	−0.000	0.018	0.007	0.004	0.009^*^
	(0.012)	(0.010)	(0.011)	(0.010)	(0.005)	(0.005)
Feels lonely (ref.: never)						
Sometimes	−0.008	0.011	−0.003	−0.023	0.006	−0.001
	(0.016)	(0.016)	(0.017)	(0.016)	(0.008)	(0.006)
Never	−0.026	−0.002	−0.034^*^	−0.031^*^	0.004	0.002
	(0.017)	(0.018)	(0.018)	(0.018)	(0.009)	(0.005)
Intercept	0.163^*^^*^^*^	0.058^*^^*^	0.097^*^^*^^*^	0.066^*^^*^^*^	0.045^*^^*^	0.005
	(0.029)	(0.025)	(0.024)	(0.024)	(0.018)	(0.010)
Individual FE	Yes	Yes	Yes	Yes	Yes	Yes
# observations	26,183	26,139	26,135	20,219	20,282	20,336

Notes: ^*^P < 0.1, ^**^P < 0.05, ^***^P < 0.01. Standard errors in parentheses robust to heteroskedasticity and individual clustering.

### Trends in informal IADL and ADL help

**Figure 1:**
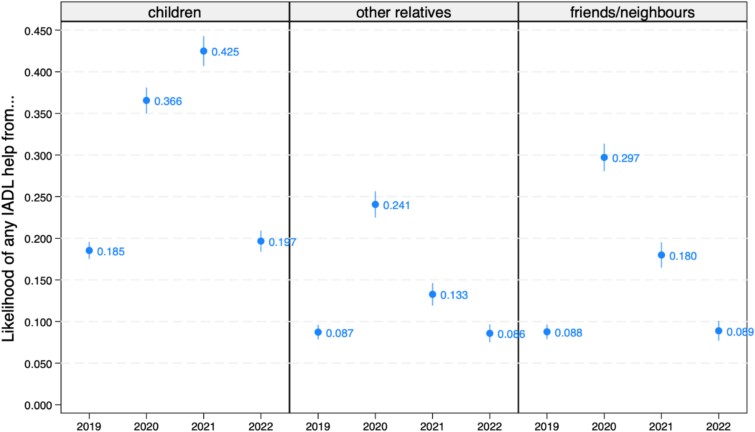
Predicted likelihoods, with 95% confidence intervals, of receiving IADL help from children, other relatives and friends/neighbours from 2019 to 2022.

We used the results from Table 2 to predict the likelihoods of receiving informal help over time, holding covariates at their means. Starting with IADL help, the likelihoods of receiving IADL support from all three sources more than doubled between 2019 and 2020 (Figure 1). The likelihood of IADL help from children increased from 18.5% in 2019 to 36.6% in 2020 and 42.5% in 2021. Compared to children, the likelihoods of IADL help from other relatives and from friends/neighbours increased more—in relative terms—between 2019 and 2020, then started to decrease in 2021. Additionally, friends and neighbours were more prevalent sources of informal IADL support during the pandemic than other relatives, despite starting at identical baseline levels. Specifically, the likelihood of IADL help from other relatives went from 8.7% to 24.1%, then down to 13.3% in 2021; the likelihood of IADL help from friends/neighbours went from 8.8% to 29.7%, then 18%. Lastly, regardless of the source, informal IADL support in 2022 was back to 2019 levels.

**Figure 2:**
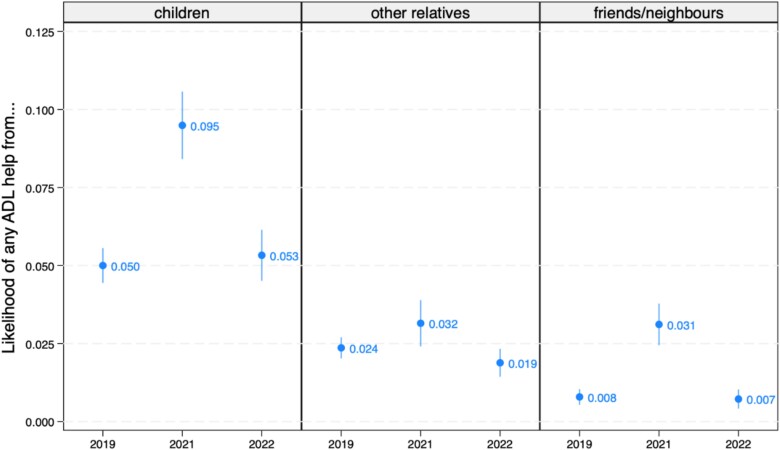
Predicted likelihoods, with 95% confidence intervals, of receiving ADL help from children, other relatives and friends/neighbours from 2019 to 2022.

ADL support showed a similar pattern over time regardless of the source, going up in 2021 compared to 2019 (2020 not available) and then back to similar levels in 2022 (Figure 2). In relative terms, friends and neighbours were the source of ADL help that increased the most, going from ˂1% in 2019 to over 3% in 2021. Yet, children remained the main providers of ADL help throughout the period, with this likelihood almost doubling from 5% in 2019 to 9.5% in 2021. There were no statistically significant differences over time for the likelihood of receiving ADL help from other relatives.

The trends in the prevalence of support were the same for those cohabiting, female living alone and male living alone. Female living alone had the highest prevalences across all three helpers, followed by male living alone. For example, during the pandemic the prevalence of IADL help from children to women living alone peaked at ˃55%, compared to ˂35% for men living alone (see Appendices 11 and 12 in the Supplementary Data file). Similarly, the estimates by country reveal similar patterns over time, with some expected variations, validating the aggregate results reported above (see Appendices 13–15 in the Supplementary Data file).

## Discussion

This is the first study that compares informal care before, during and after the COVID-19 pandemic. We shed light on how responsive various caregivers are to external, unexpected circumstances, such as a public health crisis, and the extent to which such adjustments may persist over time once the crisis is over. Our results provided three main insights. First, the likelihoods of help with IADL and ADL from both children and friends/neighbours were significantly higher during the pandemic. Second, friends and neighbours became important sources of informal support during the pandemic, with IADL help from this group becoming more prevalent than from non-children relatives. Third, the prevalences of the various kinds of support decreased back to their pre-pandemic levels by 2022.

The increased prevalences of informal support during the pandemic can be explained by various factors. The COVID-19 pandemic led governments to impose policies to control the spread of the virus, such as movement restrictions, stay-at-home requirements, suspension of community-based services (e.g. day care and in-home care) and delays to nursing home admission. Such measures, allied with greater vulnerability of older individuals to serious illness from the coronavirus, contributed to increased need for informal support. For example, many older individuals were unable to do their shopping and needed someone to do it for them (e.g. neighbours). Restricted access to formal home care had to be at least partly compensated through additional informal support, particularly from primary caregivers like children [15–17]. By 2022, provision of informal support went back to pre-pandemic levels probably because the risk of contracting the disease and its consequences were less acute, and formal LTC services were mostly available again.

Our findings are in line with the existing literature, which did not consider the post-pandemic, including three studies that also used the SHARE Corona surveys [15, 16, 24]. Bergmann and Wagner [15] took a different angle from us, focusing on 50+ caregiving individuals—thus a subset of caregivers—rather than 50+ care recipients. Still, they documented an increase in personal care provided to non-co-residing older parents during the first wave of the pandemic. Tur-Sinai *et al.* [16] reported unadjusted increases in intensity of informal help received from children, neighbours and friends, but not from other relatives, during the first wave of the pandemic. Tur-Sinai *et al.* [24] further compared informal support between 2020 and 2021 and found large variations across countries. We also found variations across countries, yet most countries showed similar trends, with prevalences of support increasing during the pandemic and returning to pre-pandemic levels by 2022.

Various studies found that non-coresident children, other relatives, and friends and neighbours are important sources of support in ‘normal times’, especially to (female) individuals residing alone where in-home support such as from a spouse is not available [5, 7–10, 32, 33]. According to Seifert and König [10], who used SHARE data for 2004–15, 4% of 50+ individuals received help from neighbours. In our study, which focused on 50+ individuals with limitations, nearly 9% of individuals received IADL support from friends/neighbours in 2019. Further, prevalence of IADL help from this group climbed to ˃29% in the first phase of the pandemic. Support from neighbours and friends often supplements informal care from family members and/or formal home care, by watching over the care receiver, helping with shopping or transportation and providing emotional support [9, 23, 34]. Such help is facilitated by proximity, accessibility and awareness of the care needs or frailty of the older persons [9]. Various other studies documented that neighbours can play a critical role during crises such as natural disasters or public health emergencies like the COVID-19 pandemic [23, 27, 28, 35, 36]. The higher prevalence of informal care from children to female living alone than to male living alone is consistent with women more easily expressing their need for help and potentially stronger relationships with children, as found for example by Kwak *et al.* [32].

The SHARE data allowed for a rich analysis of trajectories in informal support, from before to after the pandemic, from different groups of helpers, and distinguishing between IADL and ADL help. Yet, the data have a few limitations. First, as the timing of the pandemic, restrictions and vaccine rollout slightly varied across countries, our wave indicators may not capture exactly the same context across countries. Nevertheless, the periods of more stringent restrictions and the survey recall periods largely overlapped (Appendix 1). We included results by country in Appendices 13–15. Second, the recall periods may hide potentially interesting fluctuations in informal support between interviews (Appendix 1). Third, the questions on source of support included in the two Corona questionnaires already grouped children, other relatives and friends/neighbours, preventing a more granular analysis (e.g. grandchildren versus other relatives). This relationship-based grouping has good overlap with caregiver roles (e.g. primary caregivers are often children), tasks, frequency and intensity of caregiving [2, 33, 37]. Fourth, help with ADL was not asked in the first Corona Wave. Similarly, it was neither possible to study informal support with more complex medical tasks, nor with simpler companionship or emotional support. Fifth, due to lack of comparability across the Regular and Corona surveys, we reported prevalences but could not consider the intensity and frequency of informal support. We also could not explore the exact content of support (e.g. IADL and ADL support bundle a range of tasks that could not be explored in detail), quality of that support, or unmet needs of the individuals.

Two main methodological limitations are to keep in mind. First, since we captured the pandemic using time indicators, our results could partly be explained by individuals becoming frailer and needing more support over time, or other unobserved time-varying confounders. Yet, we accounted for a range of health status variables and our results are robust to various specifications that explore some of those factors (Appendices 5–10). The fact that caregiving prevalences in 2022 are similar to those observed pre-pandemic also suggest a limited role of those potential confounders. Second, we estimated linear probability models, to be able to account for individual fixed effects and adjust for unobserved time-invariant characteristics.

We found not only that the likelihoods of informal support, regardless of its type (IADL or ADL), from children and friends/neighbours significantly increased during the pandemic, but also that those likelihoods returned to their pre-pandemic levels by 2022. This shows that informal support from outside the household is responsive and adaptable to unexpected and severe events, such as a pandemic. This adaptability means that once needs have receded, support diminishes as well. In times of severe crisis, governments should be ready to provide significant support to informal caregivers who tend to adjust rapidly to changing needs [14]. Our results also point out that policies to support informal caregiving need to be tailored to different types of caregivers and targeted accordingly. From a policy perspective, this is both good and bad news. The good news is that children and friends/neighbours may respond to policies aiming at encouraging informal caregiving by increasing their informal support. The bad news is that such responses may be temporary if those policies are not significant or purposeful enough and not sustained in the long run. Help provided by friends and neighbours may offer an opportunity to partially address the LTC needs of the ageing population in Europe, but more research is needed to disentangle the causes of the variations during the pandemic and best inform policies that promote sustained caregiving [5, 10, 27]. We explored variations by living arrangement and gender of the care recipient (i.e. cohabitating, female living alone and male living alone), as well as by country (27 countries included in SHARE before, during and after the pandemic). Future research could consider variations across other relevant demographic and socioeconomic dimensions and by types of welfare states.

## Conclusion

During the COVID-19 pandemic, prevalence of informal support from various caregivers increased significantly, to return to pre-pandemic levels by 2022. Friends and neighbours played a critical role during the peak of the pandemic. Future emergency and disaster preparedness plans may want to contemplate the various forms of informal care, including support to neighbours and friends, as those helpers may be able to rapidly respond to unexpected crisis. To meet growing LTC needs in Europe, policy makers also need to consider heterogenous policies to support different types of caregivers including non-relative caregivers such as neighbours and friends.

## Supplementary Material

aa-24-1994-File002_afaf034

## Data Availability

SHARE data are publicly available upon registration here: https://share-eric.eu/data/data-access.
