# Supplementary material for: Who came to the rescue? Sources of informal support to older Europeans before, during and after the COVID-19 pandemic
Source: Age Ageing. 2025 Feb 21;54(2):afaf034. doi: 10.1093/ageing/afaf034 (PMC11843440; doi:10.1093/ageing/afaf034)
Supplement: aa-24-1994-File002_afaf034 [file aa-24-1994-file002_afaf034.docx]

**Supplementary Data for**

***Who came to the rescue?***

***Sources of informal support to older Europeans before, during,***

***and after the COVID-19 pandemic***

Table of Contents

[Appendix 1. Timeline of data collection, recall periods, and the pandemic 2](#_Toc188298339)

[Appendix 2. Sample split by country and wave 3](#_Toc188298340)

[Appendix 3. Survey questions and correspondence between informal care variables in the different waves 4](#_Toc188298341)

[Appendix 4. Sample characteristics 5](#_Toc188298342)

[Appendix 5. Full results from alternative model specifications: IADL help from children 6](#_Toc188298343)

[Appendix 6. Full results from alternative model specifications: IADL help from other relatives 8](#_Toc188298344)

[Appendix 7. Full results from alternative model specifications: IADL help from friends/neighbours 10](#_Toc188298345)

[Appendix 8. Full results from alternative model specifications: ADL help from children 12](#_Toc188298346)

[Appendix 9. Full results from alternative model specifications: ADL help from other relatives 14](#_Toc188298347)

[Appendix 10. Full results from alternative model specifications: ADL help from friends/neighbours 16](#_Toc188298348)

[Appendix 11. Predicted likelihoods, with 95% confidence intervals, of receiving IADL help from children, other relatives, and friends/neighbours from 2019 to 2022, by cohabiting status 18](#_Toc188298349)

[Appendix 12. Predicted likelihoods, with 95% confidence intervals, of receiving ADL help from children, other relatives, and friends/neighbours from 2019 to 2022, by cohabiting status 19](#_Toc188298350)

[Appendix 13. Results by country (1/3) 20](#_Toc188298351)

[Appendix 14. Results by country (2/3) 22](#_Toc188298352)

[Appendix 15. Results by country (3/3) 24](#_Toc188298353)

# Appendix 1. Timeline of data collection, recall periods, and the pandemic


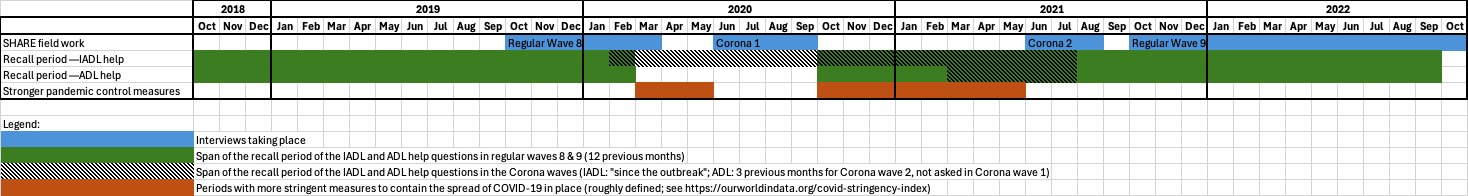


Note: the start of the pandemic and the periods with more stringent pandemic control measures are approximate, as exact dates differ across countries.

# Appendix 2. Sample split by country and wave

|  | 2019 | 2020 | 2021 | 2022 | Total |
| --- | --- | --- | --- | --- | --- |
| Austria | 354 | 241 | 208 | 228 | 1,031 |
| Germany | 651 | 382 | 252 | 408 | 1,693 |
| Sweden | 424 | 178 | 111 | 257 | 970 |
| Netherlands | 327 | 76 | 64 | 188 | 655 |
| Spain | 409 | 211 | 170 | 174 | 964 |
| Italy | 329 | 249 | 215 | 211 | 1,004 |
| France | 600 | 307 | 257 | 322 | 1,486 |
| Denmark | 360 | 205 | 142 | 198 | 905 |
| Greece | 546 | 465 | 426 | 379 | 1,816 |
| Switzerland | 273 | 192 | 170 | 176 | 811 |
| Belgium | 516 | 370 | 320 | 352 | 1,558 |
| Israel | 311 | 128 | 113 | 114 | 666 |
| Czech Republic | 644 | 356 | 284 | 343 | 1,627 |
| Poland | 519 | 241 | 224 | 337 | 1,321 |
| Luxembourg | 106 | 82 | 66 | 59 | 313 |
| Hungary | 244 | 94 | 78 | 152 | 568 |
| Slovenia | 520 | 340 | 277 | 352 | 1,489 |
| Estonia | 779 | 606 | 534 | 527 | 2,446 |
| Croatia | 321 | 170 | 152 | 212 | 855 |
| Lithuania | 286 | 211 | 182 | 199 | 878 |
| Bulgaria | 205 | 117 | 87 | 127 | 536 |
| Cyprus | 94 | 44 | 33 | 33 | 204 |
| Finland | 184 | 138 | 111 | 109 | 542 |
| Latvia | 229 | 114 | 107 | 140 | 590 |
| Malta | 65 | 49 | 40 | 41 | 195 |
| Romania | 230 | 179 | 151 | 153 | 713 |
| Slovakia | 119 | 91 | 89 | 93 | 392 |
| Total | 9,645 | 5,836 | 4,863 | 5,884 | 26,228 |

Notes: These sample sizes exclude observations with missing information on any of the covariates considered, but not on the outcome variables in order to maximize available observations. Portugal had not initiated Regular Wave 8 data collection before the COVID-19 pandemic hit and is the only SHARE participating country not represented here.

# Appendix 3. Survey questions and correspondence between informal care variables in the different waves

|  | **Regular Waves 8 & 9** | **Corona Wave 1** | **Corona Wave 2** |
| --- | --- | --- | --- |
| IADL help from   - Children - Other relatives - Friends/neighbours | *Thinking about the last 12 months, has any family member from outside the household, any friend or neighbour given you practical household help, e.g., with home repairs, gardening, transportation, shopping, household chores?* | *Since the outbreak of Corona, were you helped by others from outside of home to obtain necessities, e.g., food, medications or emergency household repairs?* | *Since the outbreak of Corona, were you helped by others from outside of home to obtain necessities, e.g., food, medications or emergency household repairs?* |
| ADL help from   - Children - Other relatives - Friends/neighbours | *Thinking about the last 12 months, has any family member from outside the household, any friend or neighbour given you personal care, e.g. dressing, bathing or showering, eating, getting in or out of bed, using the toilet?* | N.A. | *During the last three months, did you regularly receive home care provided by the following people from outside your home?* |

Notes: N.A. = not available. For the regular Waves 8 and 9, we grouped helpers (daughter, son-in-law, grandchild, sister, etc.) to match the sources of help available in the two Corona questionnaires, which were already grouped into children, other relatives, and friends/neighbours.

# Appendix 4. Sample characteristics

Out of the 9,645 individuals in the sample, 38% were observed in the four waves, 21% at baseline only, 25% at baseline and in another wave, and 17% at baseline and in two additional waves (observation patterns detailed below). Females accounted for 66% of individuals, and the average age at baseline was nearly 75 years. About 38% of individuals lived alone and 50% lived in two-person households in 2019. Of those living alone, 78% were females. These distributions were expected as we focused on persons with limitations.

| Observation pattern | #individuals | % of individuals |
| --- | --- | --- |
| 1111 | 3,649 | 38% |
| 1... | 2,006 | 21% |
| 1..1 | 1,401 | 15% |
| 11.. | 821 | 9% |
| 111. | 812 | 8% |
| 11.1 | 554 | 6% |
| 1.11 | 280 | 3% |
| 1.1. | 122 | 1% |
|  | 9,645 | 100% |

# Appendix 5. Full results from alternative model specifications: IADL help from children

|  | Model 1 | Model 2 | Model 3 | Model 4 | Model 5 |
| --- | --- | --- | --- | --- | --- |
| Year (ref.: 2019) |  |  |  |  |  |
| 2020 | 0.183*** | 0.180*** | 0.177*** | 0.171*** | 0.184*** |
|  | (0.008) | (0.012) | (0.012) | (0.011) | (0.015) |
| 2021 | 0.233*** | 0.240*** | 0.239*** | 0.235*** | 0.236*** |
|  | (0.008) | (0.012) | (0.012) | (0.012) | (0.015) |
| 2022 | 0.018*** | 0.011 | 0.011 | 0.010 | 0.014 |
|  | (0.006) | (0.009) | (0.009) | (0.009) | (0.012) |
| Ability to make ends meet (ref.: with great difficulty) | |  |  |  |  |
| With some difficulty | 0.020* | 0.017 | 0.016 | 0.011 | 0.016 |
|  | (0.012) | (0.019) | (0.019) | (0.019) | (0.025) |
| Fairly easily | 0.010 | 0.035 | 0.035 | 0.025 | 0.038 |
|  | (0.014) | (0.022) | (0.022) | (0.022) | (0.028) |
| Easily | 0.005 | 0.024 | 0.024 | 0.014 | 0.013 |
|  | (0.016) | (0.024) | (0.024) | (0.024) | (0.030) |
| Self-assessed health (ref.: poor) | |  |  |  |  |
| Fair | -0.008 | -0.010 | -0.009 |  | -0.015 |
|  | (0.009) | (0.016) | (0.016) |  | (0.021) |
| Good | -0.025** | -0.023 | -0.022 |  | -0.012 |
|  | (0.012) | (0.020) | (0.020) |  | (0.026) |
| Very good | -0.037** | -0.043 | -0.043 |  | -0.063** |
|  | (0.018) | (0.026) | (0.026) |  | (0.032) |
| Excellent | -0.029 | 0.017 | 0.018 |  | -0.015 |
|  | (0.032) | (0.039) | (0.039) |  | (0.051) |
| Drugs for: |  |  |  |  |  |
| High cholesterol | -0.012 | -0.018 | -0.018 |  | -0.006 |
|  | (0.010) | (0.015) | (0.015) |  | (0.019) |
| High blood pressure | 0.013 | -0.001 | -0.002 |  | 0.015 |
|  | (0.011) | (0.016) | (0.016) |  | (0.021) |
| Coronary diseases | 0.023** | 0.023 | 0.022 |  | 0.031 |
|  | (0.010) | (0.016) | (0.016) |  | (0.019) |
| Other heart diseases | 0.003 | -0.004 | -0.004 |  | -0.007 |
|  | (0.009) | (0.015) | (0.015) |  | (0.019) |
| Diabetes | 0.008 | -0.024 | -0.023 |  | 0.004 |
|  | (0.015) | (0.023) | (0.022) |  | (0.031) |
| Chronic bronchitis | 0.017 | 0.001 | 0.002 |  | 0.026 |
|  | (0.016) | (0.025) | (0.025) |  | (0.032) |
| Frailty symptoms |  |  |  |  |  |
| Falls | 0.004 | 0.005 | 0.005 |  | 0.011 |
|  | (0.009) | (0.016) | (0.016) |  | (0.022) |
| Fear of falling | 0.013 | 0.010 | 0.009 |  | 0.019 |
|  | (0.008) | (0.014) | (0.014) |  | (0.018) |
| Dizziness | 0.025*** | 0.031** | 0.030** |  | 0.035** |
|  | (0.008) | (0.013) | (0.013) |  | (0.015) |
| Fatigue | 0.023*** | 0.004 | 0.004 |  | -0.001 |
|  | (0.008) | (0.013) | (0.013) |  | (0.016) |
| Sad or depressed | 0.029*** | 0.044*** | 0.043*** |  | 0.020 |
|  | (0.007) | (0.012) | (0.012) |  | (0.015) |
| Sleeping troubles | -0.001 | -0.009 | -0.009 |  | -0.018 |
|  | (0.008) | (0.012) | (0.012) |  | (0.016) |
| Feels lonely (ref.: never) |  |  |  |  |  |
| Sometimes | -0.018 | -0.008 | -0.007 |  | -0.030 |
|  | (0.011) | (0.016) | (0.016) |  | (0.021) |
| Never | -0.022* | -0.026 | -0.025 |  | -0.038* |
|  | (0.012) | (0.017) | (0.017) |  | (0.022) |
| Difficulties accessing home care | |  | 0.075* |  |  |
|  |  |  | (0.041) |  |  |
| Intercept | 0.187*** | 0.163*** | 0.163*** | 0.174*** | 0.163*** |
|  | (0.019) | (0.029) | (0.029) | (0.017) | (0.039) |
| Individual FE | Yes | Yes | Yes | Yes | Yes |
| Weighted analysis | No | Yes | Yes | Yes | Yes |
| Balanced panel | No | No | No | No | Yes |
| # observations | 26,183 | 26,183 | 26,183 | 26,183 | 14,577 |

Notes: *p<0.1, **p<0.05, ***p<0.01. Standard errors in parentheses robust to heteroskedasticity and individual clustering. Model 2 is the one used to predict the mean likelihoods of receiving help reported in the main text.

# Appendix 6. Full results from alternative model specifications: IADL help from other relatives

|  | Model 1 | Model 2 | Model 3 | Model 4 | Model 5 |
| --- | --- | --- | --- | --- | --- |
| Year (ref.: 2019) |  |  |  |  |  |
| 2020 | 0.142*** | 0.153*** | 0.149*** | 0.145*** | 0.151*** |
|  | (0.007) | (0.011) | (0.012) | (0.010) | (0.014) |
| 2021 | 0.012** | 0.045*** | 0.044*** | 0.042*** | 0.043*** |
|  | (0.006) | (0.009) | (0.009) | (0.009) | (0.011) |
| 2022 | 0.006 | -0.001 | -0.002 | -0.003 | 0.000 |
|  | (0.005) | (0.008) | (0.008) | (0.008) | (0.009) |
| Ability to make ends meet (ref.: with great difficulty) | |  |  |  |  |
| With some difficulty | 0.016* | 0.011 | 0.010 | 0.007 | -0.009 |
|  | (0.010) | (0.016) | (0.016) | (0.017) | (0.020) |
| Fairly easily | 0.024** | 0.043** | 0.043** | 0.035* | 0.016 |
|  | (0.011) | (0.019) | (0.019) | (0.019) | (0.022) |
| Easily | 0.037*** | 0.053** | 0.053** | 0.044** | 0.009 |
|  | (0.013) | (0.021) | (0.021) | (0.021) | (0.026) |
| Self-assessed health (ref.: poor) | |  |  |  |  |
| Fair | -0.019** | -0.019 | -0.018 |  | -0.019 |
|  | (0.008) | (0.013) | (0.013) |  | (0.017) |
| Good | -0.035*** | -0.037** | -0.037** |  | -0.037* |
|  | (0.010) | (0.016) | (0.016) |  | (0.021) |
| Very good | -0.030** | -0.054** | -0.053** |  | -0.041 |
|  | (0.014) | (0.023) | (0.023) |  | (0.028) |
| Excellent | -0.057** | -0.070* | -0.068* |  | -0.037 |
|  | (0.022) | (0.039) | (0.039) |  | (0.038) |
| Drugs for: |  |  |  |  |  |
| High cholesterol | -0.012 | -0.027** | -0.027** |  | -0.022 |
|  | (0.008) | (0.013) | (0.013) |  | (0.017) |
| High blood pressure | -0.005 | 0.002 | 0.001 |  | 0.002 |
|  | (0.009) | (0.013) | (0.013) |  | (0.017) |
| Coronary diseases | 0.001 | -0.012 | -0.012 |  | -0.011 |
|  | (0.008) | (0.015) | (0.015) |  | (0.019) |
| Other heart diseases | -0.008 | -0.007 | -0.007 |  | 0.006 |
|  | (0.008) | (0.013) | (0.013) |  | (0.018) |
| Diabetes | 0.021* | 0.020 | 0.020 |  | 0.002 |
|  | (0.012) | (0.023) | (0.023) |  | (0.032) |
| Chronic bronchitis | 0.035** | 0.052** | 0.053** |  | 0.034 |
|  | (0.015) | (0.025) | (0.025) |  | (0.029) |
| Frailty symptoms |  |  |  |  |  |
| Falls | -0.010 | 0.007 | 0.007 |  | 0.002 |
|  | (0.007) | (0.014) | (0.014) |  | (0.018) |
| Fear of falling | 0.015** | 0.012 | 0.012 |  | 0.003 |
|  | (0.007) | (0.012) | (0.012) |  | (0.015) |
| Dizziness | 0.011 | 0.020* | 0.019* |  | 0.024* |
|  | (0.007) | (0.011) | (0.011) |  | (0.013) |
| Fatigue | 0.004 | -0.003 | -0.004 |  | -0.001 |
|  | (0.006) | (0.011) | (0.011) |  | (0.013) |
| Sad or depressed | 0.011* | 0.020** | 0.020** |  | 0.012 |
|  | (0.006) | (0.010) | (0.010) |  | (0.012) |
| Sleeping troubles | 0.001 | -0.000 | 0.000 |  | 0.000 |
|  | (0.006) | (0.010) | (0.010) |  | (0.012) |
| Feels lonely (ref.: never) |  |  |  |  |  |
| Sometimes | -0.001 | 0.011 | 0.012 |  | 0.003 |
|  | (0.010) | (0.016) | (0.016) |  | (0.019) |
| Never | -0.003 | -0.002 | -0.001 |  | -0.005 |
|  | (0.011) | (0.018) | (0.017) |  | (0.021) |
| Difficulties accessing home care | |  | 0.125*** |  |  |
|  |  |  | (0.045) |  |  |
| Intercept | 0.081*** | 0.058** | 0.058** | 0.066*** | 0.087*** |
|  | (0.016) | (0.025) | (0.025) | (0.015) | (0.032) |
| Individual FE | Yes | Yes | Yes | Yes | Yes |
| Weighted analysis | No | Yes | Yes | Yes | Yes |
| Balanced panel | No | No | No | No | Yes |
| # observations | 26,139 | 26,139 | 26,139 | 26,139 | 14,539 |

Notes: *p<0.1, **p<0.05, ***p<0.01. Standard errors in parentheses robust to heteroskedasticity and individual clustering. Model 2 is the one used to predict the mean likelihoods of receiving help reported in the main text.

# Appendix 7. Full results from alternative model specifications: IADL help from friends/neighbours

|  | Model 1 | Model 2 | Model 3 | Model 4 | Model 5 |
| --- | --- | --- | --- | --- | --- |
| Year (ref.: 2019) |  |  |  |  |  |
| 2020 | 0.214*** | 0.209*** | 0.204*** | 0.194*** | 0.213*** |
|  | (0.007) | (0.011) | (0.011) | (0.011) | (0.015) |
| 2021 | 0.077*** | 0.092*** | 0.090*** | 0.083*** | 0.089*** |
|  | (0.006) | (0.010) | (0.010) | (0.010) | (0.012) |
| 2022 | 0.004 | 0.001 | 0.001 | -0.002 | 0.012 |
|  | (0.005) | (0.008) | (0.008) | (0.009) | (0.009) |
| Ability to make ends meet (ref.: with great difficulty) | |  |  |  |  |
| With some difficulty | 0.001 | 0.014 | 0.014 | 0.008 | 0.013 |
|  | (0.010) | (0.016) | (0.016) | (0.016) | (0.019) |
| Fairly easily | -0.006 | 0.026 | 0.026 | 0.013 | 0.021 |
|  | (0.012) | (0.019) | (0.019) | (0.019) | (0.023) |
| Easily | -0.002 | 0.020 | 0.020 | 0.006 | 0.014 |
|  | (0.013) | (0.022) | (0.022) | (0.022) | (0.028) |
| Self-assessed health (ref.: poor) | |  |  |  |  |
| Fair | -0.028*** | -0.028** | -0.028** |  | -0.024 |
|  | (0.008) | (0.013) | (0.013) |  | (0.016) |
| Good | -0.048*** | -0.053*** | -0.053*** |  | -0.054** |
|  | (0.010) | (0.017) | (0.017) |  | (0.022) |
| Very good | -0.059*** | -0.100*** | -0.099*** |  | -0.087*** |
|  | (0.015) | (0.025) | (0.025) |  | (0.031) |
| Excellent | -0.069*** | -0.092** | -0.091* |  | -0.074 |
|  | (0.026) | (0.047) | (0.046) |  | (0.055) |
| Drugs for: |  |  |  |  |  |
| High cholesterol | -0.009 | -0.038** | -0.038** |  | -0.024 |
|  | (0.008) | (0.015) | (0.015) |  | (0.017) |
| High blood pressure | 0.007 | 0.004 | 0.003 |  | 0.008 |
|  | (0.009) | (0.014) | (0.014) |  | (0.017) |
| Coronary diseases | 0.008 | 0.018 | 0.017 |  | 0.003 |
|  | (0.008) | (0.014) | (0.014) |  | (0.017) |
| Other heart diseases | -0.002 | 0.001 | 0.001 |  | -0.005 |
|  | (0.008) | (0.012) | (0.012) |  | (0.015) |
| Diabetes | -0.015 | -0.011 | -0.011 |  | -0.038 |
|  | (0.012) | (0.024) | (0.024) |  | (0.030) |
| Chronic bronchitis | 0.020 | 0.013 | 0.014 |  | 0.019 |
|  | (0.014) | (0.024) | (0.024) |  | (0.027) |
| Frailty symptoms |  |  |  |  |  |
| Falls | 0.004 | 0.011 | 0.011 |  | 0.016 |
|  | (0.007) | (0.013) | (0.013) |  | (0.016) |
| Fear of falling | 0.001 | 0.011 | 0.011 |  | 0.010 |
|  | (0.007) | (0.012) | (0.012) |  | (0.014) |
| Dizziness | 0.003 | 0.013 | 0.013 |  | 0.024* |
|  | (0.007) | (0.011) | (0.011) |  | (0.014) |
| Fatigue | 0.009 | -0.017 | -0.017 |  | -0.012 |
|  | (0.006) | (0.011) | (0.011) |  | (0.013) |
| Sad or depressed | 0.016** | 0.035*** | 0.034*** |  | 0.020 |
|  | (0.006) | (0.011) | (0.011) |  | (0.012) |
| Sleeping troubles | 0.013** | 0.018 | 0.018 |  | 0.014 |
|  | (0.006) | (0.011) | (0.011) |  | (0.013) |
| Feels lonely (ref.: never) |  |  |  |  |  |
| Sometimes | -0.018* | -0.003 | -0.001 |  | -0.008 |
|  | (0.010) | (0.017) | (0.017) |  | (0.020) |
| Never | -0.035*** | -0.034* | -0.033* |  | -0.023 |
|  | (0.010) | (0.018) | (0.018) |  | (0.021) |
| Difficulties accessing home care | |  | 0.143*** |  |  |
|  |  |  | (0.054) |  |  |
| Intercept | 0.116*** | 0.097*** | 0.097*** | 0.085*** | 0.093*** |
|  | (0.016) | (0.024) | (0.024) | (0.014) | (0.029) |
| Individual FE | Yes | Yes | Yes | Yes | Yes |
| Weighted analysis | No | Yes | Yes | Yes | Yes |
| Balanced panel | No | No | No | No | Yes |
| # observations | 26,135 | 26,135 | 26,135 | 26,135 | 14,541 |

Notes: *p<0.1, **p<0.05, ***p<0.01. Standard errors in parentheses robust to heteroskedasticity and individual clustering. Model 2 is the one used to predict the mean likelihoods of receiving help reported in the main text.

# Appendix 8. Full results from alternative model specifications: ADL help from children

|  | Model 1 | Model 2 | Model 3 | Model 4 | Model 5 |
| --- | --- | --- | --- | --- | --- |
| Year (ref.: 2019) |  |  |  |  |  |
| 2021 | 0.040*** | 0.045*** | 0.042*** | 0.042*** | 0.038*** |
|  | (0.005) | (0.007) | (0.007) | (0.008) | (0.008) |
| 2022 | 0.011*** | 0.003 | 0.003 | 0.003 | 0.010* |
|  | (0.003) | (0.006) | (0.006) | (0.007) | (0.006) |
| Ability to make ends meet (ref.: with great difficulty) | |  |  |  |  |
| With some difficulty | 0.002 | -0.007 | -0.007 | -0.011 | -0.012 |
|  | (0.009) | (0.018) | (0.018) | (0.019) | (0.024) |
| Fairly easily | -0.006 | -0.004 | -0.005 | -0.011 | -0.011 |
|  | (0.010) | (0.018) | (0.018) | (0.018) | (0.023) |
| Easily | -0.002 | -0.014 | -0.014 | -0.021 | -0.020 |
|  | (0.011) | (0.019) | (0.019) | (0.019) | (0.024) |
| Self-assessed health (ref.: poor) | |  |  |  |  |
| Fair | -0.019** | -0.015 | -0.014 |  | -0.024 |
|  | (0.007) | (0.012) | (0.012) |  | (0.016) |
| Good | -0.021** | -0.013 | -0.013 |  | -0.029* |
|  | (0.009) | (0.013) | (0.013) |  | (0.016) |
| Very good | -0.031*** | -0.034** | -0.034** |  | -0.054*** |
|  | (0.012) | (0.017) | (0.017) |  | (0.020) |
| Excellent | -0.021 | -0.014 | -0.014 |  | -0.039* |
|  | (0.015) | (0.019) | (0.018) |  | (0.023) |
| Drugs for: |  |  |  |  |  |
| High cholesterol | -0.006 | -0.030** | -0.030** |  | -0.023* |
|  | (0.007) | (0.015) | (0.015) |  | (0.012) |
| High blood pressure | 0.004 | 0.005 | 0.004 |  | 0.006 |
|  | (0.007) | (0.009) | (0.009) |  | (0.011) |
| Coronary diseases | 0.012 | 0.015 | 0.013 |  | 0.033** |
|  | (0.007) | (0.012) | (0.012) |  | (0.014) |
| Other heart diseases | 0.015** | 0.001 | 0.002 |  | 0.011 |
|  | (0.007) | (0.011) | (0.011) |  | (0.012) |
| Diabetes | 0.003 | 0.010 | 0.009 |  | 0.010 |
|  | (0.011) | (0.018) | (0.018) |  | (0.023) |
| Chronic bronchitis | -0.012 | -0.011 | -0.010 |  | -0.018 |
|  | (0.012) | (0.016) | (0.015) |  | (0.019) |
| Frailty symptoms |  |  |  |  |  |
| Falls | 0.020*** | 0.014 | 0.012 |  | 0.025** |
|  | (0.007) | (0.011) | (0.011) |  | (0.013) |
| Fear of falling | 0.005 | 0.011 | 0.011 |  | 0.011 |
|  | (0.005) | (0.011) | (0.011) |  | (0.013) |
| Dizziness | 0.015*** | 0.013 | 0.013 |  | 0.012 |
|  | (0.005) | (0.009) | (0.009) |  | (0.009) |
| Fatigue | 0.002 | -0.001 | -0.001 |  | -0.008 |
|  | (0.005) | (0.010) | (0.010) |  | (0.013) |
| Sad or depressed | 0.010** | 0.022** | 0.023** |  | 0.003 |
|  | (0.005) | (0.009) | (0.009) |  | (0.009) |
| Sleeping troubles | 0.008 | 0.007 | 0.006 |  | -0.006 |
|  | (0.005) | (0.010) | (0.010) |  | (0.010) |
| Feels lonely (ref.: never) |  |  |  |  |  |
| Sometimes | -0.018** | -0.023 | -0.021 |  | -0.003 |
|  | (0.009) | (0.016) | (0.015) |  | (0.015) |
| Never | -0.016* | -0.031* | -0.029 |  | -0.012 |
|  | (0.009) | (0.018) | (0.018) |  | (0.014) |
| Difficulties accessing home care | |  | 0.308*** |  |  |
|  |  |  | (0.092) |  |  |
| Intercept | 0.048*** | 0.066*** | 0.066*** | 0.063*** | 0.065** |
|  | (0.014) | (0.024) | (0.024) | (0.015) | (0.031) |
| Individual FE | Yes | Yes | Yes | Yes | Yes |
| Weighted analysis | No | Yes | Yes | Yes | Yes |
| Balanced panel | No | No | No | No | Yes |
| # observations | 20,219 | 20,219 | 20,219 | 20,219 | 10,835 |

Notes: *p<0.1, **p<0.05, ***p<0.01. Standard errors in parentheses robust to heteroskedasticity and individual clustering. Model 2 is the one used to predict the mean likelihoods of receiving help reported in the main text.

# Appendix 9. Full results from alternative model specifications: ADL help from other relatives

|  | Model 1 | Model 2 | Model 3 | Model 4 | Model 5 |
| --- | --- | --- | --- | --- | --- |
| Year (ref.: 2019) |  |  |  |  |  |
| 2021 | 0.003 | 0.008 | 0.006 | 0.005 | 0.006 |
|  | (0.003) | (0.005) | (0.005) | (0.006) | (0.005) |
| 2022 | -0.003 | -0.005 | -0.005 | -0.006* | -0.007* |
|  | (0.002) | (0.003) | (0.003) | (0.003) | (0.004) |
| Ability to make ends meet (ref.: with great difficulty) | |  |  |  |  |
| With some difficulty | -0.003 | -0.016 | -0.016 | -0.020 | -0.020 |
|  | (0.005) | (0.014) | (0.014) | (0.014) | (0.020) |
| Fairly easily | 0.000 | -0.008 | -0.008 | -0.014 | -0.010 |
|  | (0.005) | (0.012) | (0.012) | (0.013) | (0.018) |
| Easily | 0.001 | -0.007 | -0.008 | -0.015 | -0.010 |
|  | (0.006) | (0.015) | (0.015) | (0.015) | (0.019) |
| Self-assessed health (ref.: poor) | |  |  |  |  |
| Fair | -0.014*** | -0.025*** | -0.025*** |  | -0.031*** |
|  | (0.005) | (0.009) | (0.009) |  | (0.012) |
| Good | -0.018*** | -0.033*** | -0.032*** |  | -0.032*** |
|  | (0.006) | (0.010) | (0.010) |  | (0.011) |
| Very good | -0.019** | -0.014 | -0.014 |  | -0.009 |
|  | (0.008) | (0.023) | (0.023) |  | (0.033) |
| Excellent | -0.014 | -0.020 | -0.019 |  | -0.024 |
|  | (0.010) | (0.013) | (0.013) |  | (0.019) |
| Drugs for: |  |  |  |  |  |
| High cholesterol | -0.000 | -0.008 | -0.008 |  | -0.004 |
|  | (0.004) | (0.006) | (0.006) |  | (0.007) |
| High blood pressure | -0.010** | -0.009 | -0.009 |  | -0.010 |
|  | (0.004) | (0.009) | (0.009) |  | (0.009) |
| Coronary diseases | -0.007* | -0.017** | -0.017** |  | -0.016* |
|  | (0.004) | (0.007) | (0.007) |  | (0.008) |
| Other heart diseases | 0.001 | -0.001 | -0.001 |  | 0.003 |
|  | (0.004) | (0.008) | (0.008) |  | (0.010) |
| Diabetes | 0.002 | 0.015 | 0.015 |  | 0.016 |
|  | (0.007) | (0.012) | (0.012) |  | (0.020) |
| Chronic bronchitis | 0.002 | 0.017 | 0.017 |  | 0.012 |
|  | (0.007) | (0.016) | (0.016) |  | (0.021) |
| Frailty symptoms |  |  |  |  |  |
| Falls | 0.009** | 0.018** | 0.017* |  | 0.023* |
|  | (0.004) | (0.009) | (0.009) |  | (0.013) |
| Fear of falling | -0.002 | -0.013* | -0.013* |  | -0.016 |
|  | (0.003) | (0.007) | (0.007) |  | (0.010) |
| Dizziness | 0.007** | 0.011* | 0.011* |  | 0.008 |
|  | (0.003) | (0.006) | (0.006) |  | (0.007) |
| Fatigue | 0.005* | 0.006 | 0.006 |  | 0.001 |
|  | (0.003) | (0.007) | (0.007) |  | (0.009) |
| Sad or depressed | -0.000 | 0.005 | 0.005 |  | 0.009 |
|  | (0.003) | (0.005) | (0.005) |  | (0.006) |
| Sleeping troubles | 0.003 | 0.004 | 0.004 |  | 0.003 |
|  | (0.003) | (0.005) | (0.005) |  | (0.006) |
| Feels lonely (ref.: never) |  |  |  |  |  |
| Sometimes | -0.002 | 0.006 | 0.007 |  | 0.001 |
|  | (0.005) | (0.008) | (0.008) |  | (0.009) |
| Never | -0.002 | 0.004 | 0.004 |  | -0.001 |
|  | (0.005) | (0.009) | (0.009) |  | (0.011) |
| Difficulties accessing home care | |  | 0.136* |  |  |
|  |  |  | (0.072) |  |  |
| Intercept | 0.034*** | 0.045** | 0.045** | 0.038*** | 0.052** |
|  | (0.008) | (0.018) | (0.018) | (0.011) | (0.026) |
| Individual FE | Yes | Yes | Yes | Yes | Yes |
| Weighted analysis | No | Yes | Yes | Yes | Yes |
| Balanced panel | No | No | No | No | Yes |
| # observations | 20,282 | 20,282 | 20,282 | 20,282 | 10,887 |

Notes: *p<0.1, **p<0.05, ***p<0.01. Standard errors in parentheses robust to heteroskedasticity and individual clustering. Model 2 is the one used to predict the mean likelihoods of receiving help reported in the main text.

# Appendix 10. Full results from alternative model specifications: ADL help from friends/neighbours

|  | Model 1 | Model 2 | Model 3 | Model 4 | Model 5 |
| --- | --- | --- | --- | --- | --- |
| Year (ref.: 2019) |  |  |  |  |  |
| 2021 | 0.017*** | 0.023*** | 0.021*** | 0.021*** | 0.021*** |
|  | (0.003) | (0.004) | (0.004) | (0.004) | (0.005) |
| 2022 | 0.000 | -0.001 | -0.001 | -0.002 | 0.000 |
|  | (0.002) | (0.002) | (0.002) | (0.002) | (0.002) |
| Ability to make ends meet (ref.: with great difficulty) | |  |  |  |  |
| With some difficulty | 0.001 | 0.008 | 0.008 | 0.006 | 0.011 |
|  | (0.005) | (0.007) | (0.007) | (0.006) | (0.008) |
| Fairly easily | 0.004 | 0.015** | 0.015* | 0.013* | 0.018* |
|  | (0.006) | (0.008) | (0.008) | (0.007) | (0.010) |
| Easily | 0.011* | 0.021** | 0.020** | 0.019** | 0.027** |
|  | (0.006) | (0.009) | (0.009) | (0.008) | (0.012) |
| Self-assessed health (ref.: poor) | |  |  |  |  |
| Fair | -0.004 | -0.010 | -0.009 |  | -0.009 |
|  | (0.004) | (0.007) | (0.007) |  | (0.009) |
| Good | -0.005 | -0.013* | -0.012 |  | -0.011 |
|  | (0.005) | (0.007) | (0.007) |  | (0.009) |
| Very good | -0.002 | -0.011 | -0.010 |  | 0.001 |
|  | (0.006) | (0.014) | (0.014) |  | (0.017) |
| Excellent | -0.007 | -0.009 | -0.009 |  | -0.004 |
|  | (0.006) | (0.010) | (0.009) |  | (0.012) |
| Drugs for: |  |  |  |  |  |
| High cholesterol | -0.002 | -0.009 | -0.008 |  | -0.007 |
|  | (0.003) | (0.006) | (0.006) |  | (0.006) |
| High blood pressure | -0.008** | -0.012** | -0.013** |  | -0.013 |
|  | (0.004) | (0.006) | (0.006) |  | (0.008) |
| Coronary diseases | 0.002 | -0.003 | -0.003 |  | 0.002 |
|  | (0.003) | (0.006) | (0.006) |  | (0.006) |
| Other heart diseases | -0.001 | -0.001 | -0.001 |  | 0.004 |
|  | (0.004) | (0.007) | (0.007) |  | (0.009) |
| Diabetes | 0.005 | 0.004 | 0.003 |  | 0.017 |
|  | (0.005) | (0.011) | (0.011) |  | (0.017) |
| Chronic bronchitis | -0.001 | 0.004 | 0.004 |  | 0.023 |
|  | (0.005) | (0.014) | (0.014) |  | (0.018) |
| Frailty symptoms |  |  |  |  |  |
| Falls | 0.003 | 0.001 | -0.001 |  | 0.001 |
|  | (0.003) | (0.005) | (0.005) |  | (0.005) |
| Fear of falling | 0.004 | 0.002 | 0.002 |  | 0.006 |
|  | (0.003) | (0.005) | (0.005) |  | (0.005) |
| Dizziness | 0.002 | 0.004 | 0.004 |  | 0.003 |
|  | (0.003) | (0.005) | (0.005) |  | (0.006) |
| Fatigue | -0.002 | -0.003 | -0.003 |  | -0.004 |
|  | (0.003) | (0.004) | (0.004) |  | (0.006) |
| Sad or depressed | 0.002 | 0.004 | 0.004 |  | 0.000 |
|  | (0.003) | (0.005) | (0.005) |  | (0.005) |
| Sleeping troubles | 0.005** | 0.009* | 0.008 |  | 0.011* |
|  | (0.003) | (0.005) | (0.005) |  | (0.007) |
| Feels lonely (ref.: never) |  |  |  |  |  |
| Sometimes | -0.002 | -0.001 | -0.000 |  | -0.001 |
|  | (0.004) | (0.006) | (0.006) |  | (0.007) |
| Never | 0.001 | 0.002 | 0.002 |  | 0.006 |
|  | (0.004) | (0.005) | (0.005) |  | (0.006) |
| Difficulties accessing home care | |  | 0.206** |  |  |
|  |  |  | (0.082) |  |  |
| Intercept | 0.006 | 0.005 | 0.005 | -0.002 | -0.009 |
|  | (0.008) | (0.010) | (0.010) | (0.006) | (0.012) |
| Individual FE | Yes | Yes | Yes | Yes | Yes |
| Weighted analysis | No | Yes | Yes | Yes | Yes |
| Balanced panel | No | No | No | No | Yes |
| # observations | 20,336 | 20,336 | 20,336 | 20,336 | 10,920 |

Notes: *p<0.1, **p<0.05, ***p<0.01. Standard errors in parentheses robust to heteroskedasticity and individual clustering. Model 2 is the one used to predict the mean likelihoods of receiving help reported in the main text.


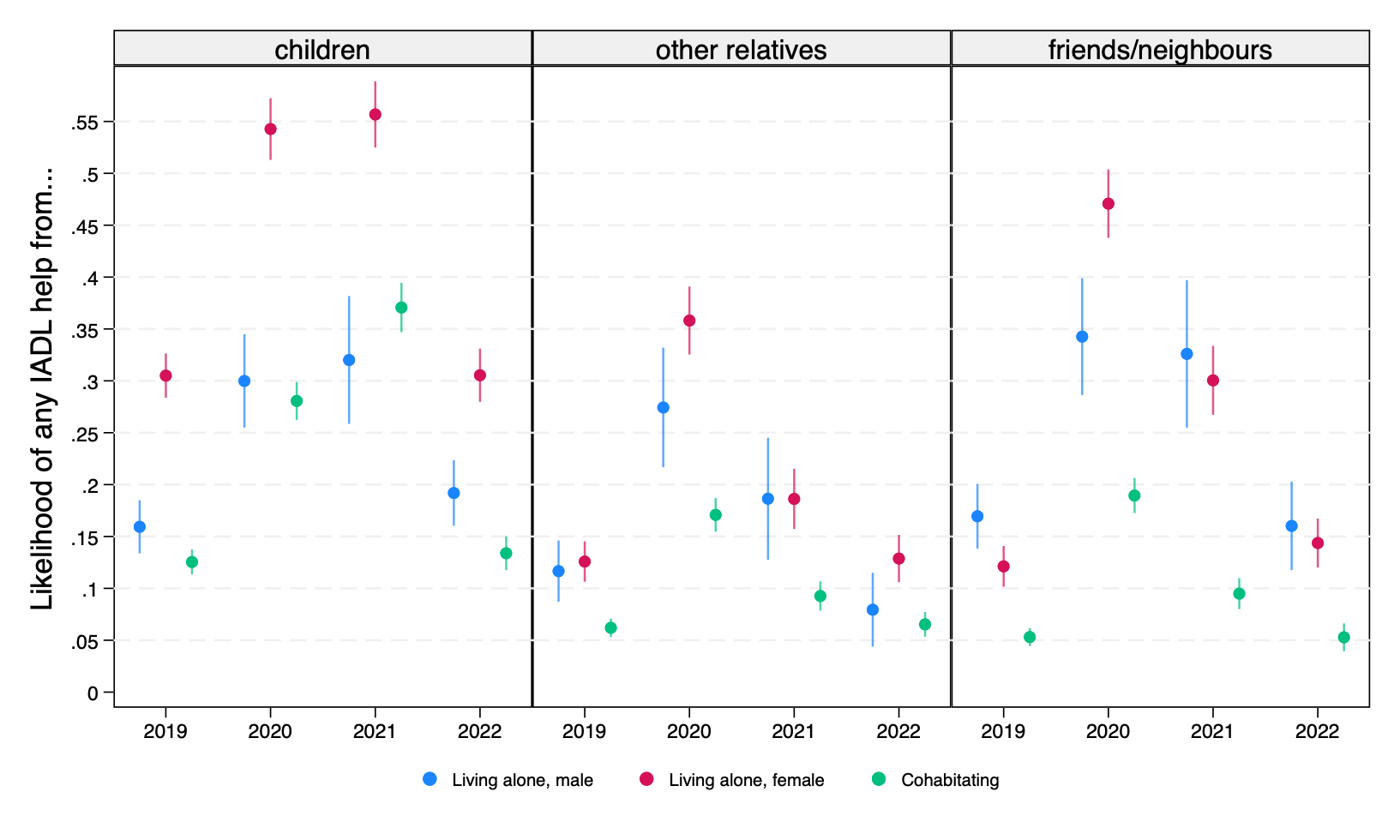


# Appendix 11. Predicted likelihoods, with 95% confidence intervals, of receiving IADL help from children, other relatives, and friends/neighbours from 2019 to 2022, by cohabiting status


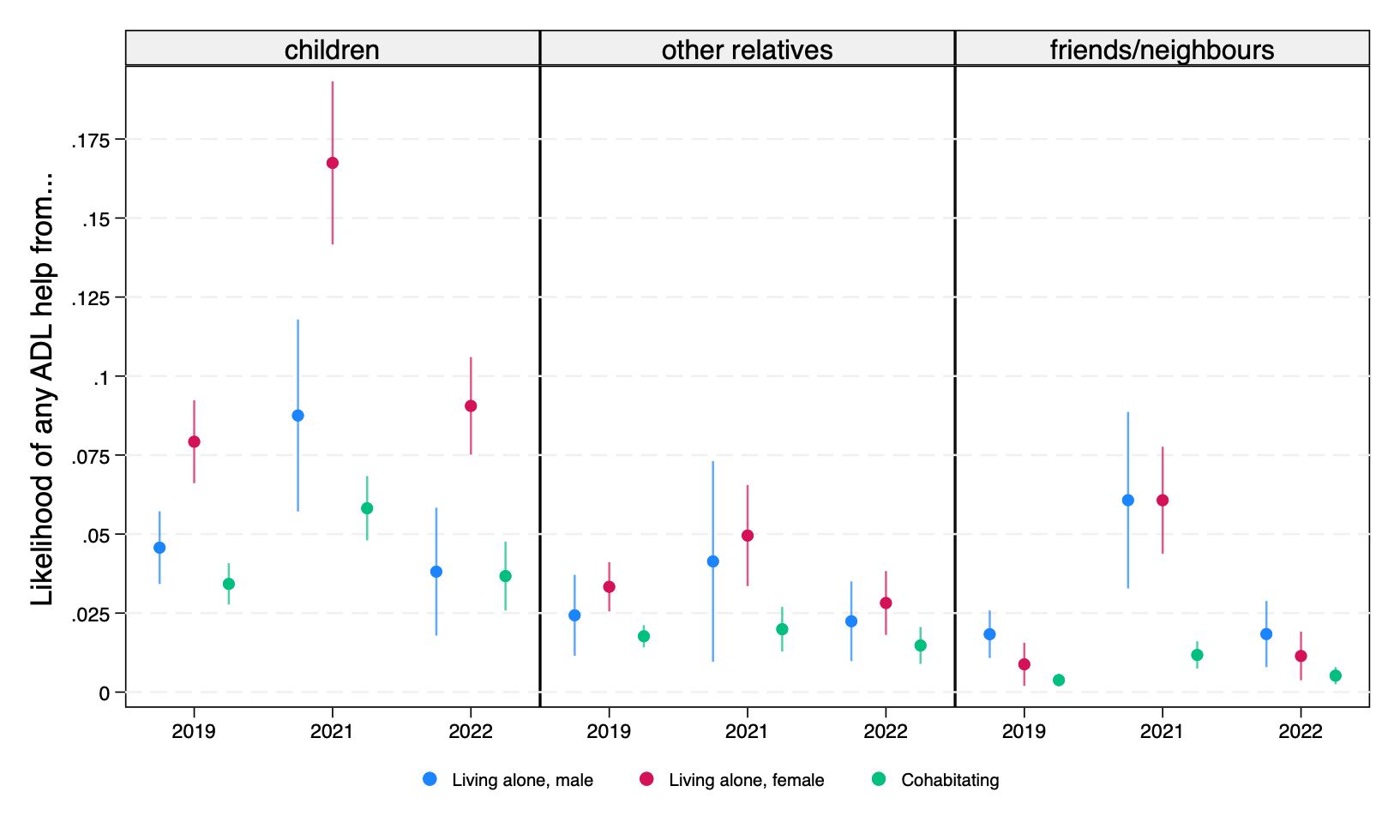


# Appendix 12. Predicted likelihoods, with 95% confidence intervals, of receiving ADL help from children, other relatives, and friends/neighbours from 2019 to 2022, by cohabiting status

# Appendix 13. Results by country (1/3)

|  | Austria | Belgium | Bulgaria | Croatia | Cyprus | Czech Republic | Denmark | Estonia | Finland |
| --- | --- | --- | --- | --- | --- | --- | --- | --- | --- |
|  | IADL help from children | | | | | | | | |
| Year (ref.: 2019) | |  |  |  |  |  |  |  |  |
| 2020 | 0.263*** | 0.194*** | 0.225*** | 0.175*** | 0.453*** | -0.032 | 0.022 | 0.048* | 0.212*** |
|  | (0.042) | (0.031) | (0.060) | (0.048) | (0.093) | (0.037) | (0.039) | (0.027) | (0.059) |
| 2021 | 0.201*** | 0.182*** | 0.449*** | 0.308*** | 0.318*** | 0.048 | -0.016 | 0.149*** | 0.244*** |
|  | (0.048) | (0.031) | (0.068) | (0.046) | (0.101) | (0.044) | (0.039) | (0.029) | (0.070) |
| 2022 | 0.061 | 0.020 | 0.031 | -0.014 | 0.036 | 0.012 | -0.027 | -0.031 | 0.082 |
|  | (0.038) | (0.025) | (0.051) | (0.032) | (0.085) | (0.034) | (0.034) | (0.024) | (0.050) |
| # observations | 1,030 | 1,557 | 533 | 852 | 204 | 1,624 | 904 | 2,445 | 540 |
|  | IADL help from other relatives | | | | | | | | |
| Year (ref.: 2019) | |  |  |  |  |  |  |  |  |
| 2020 | 0.215*** | 0.082*** | 0.175*** | 0.144*** | 0.075* | 0.026 | 0.085** | 0.088*** | 0.107** |
|  | (0.044) | (0.026) | (0.043) | (0.037) | (0.044) | (0.028) | (0.036) | (0.024) | (0.053) |
| 2021 | 0.048 | -0.012 | 0.101** | -0.071** | 0.106* | -0.058** | -0.035 | -0.063*** | -0.083** |
|  | (0.032) | (0.025) | (0.047) | (0.033) | (0.054) | (0.026) | (0.030) | (0.021) | (0.036) |
| 2022 | 0.011 | 0.005 | 0.101*** | 0.009 | 0.145** | -0.033 | 0.058** | 0.008 | -0.057 |
|  | (0.024) | (0.024) | (0.036) | (0.025) | (0.070) | (0.022) | (0.025) | (0.018) | (0.036) |
| # observations | 1,027 | 1,557 | 532 | 849 | 204 | 1,622 | 901 | 2,442 | 537 |
|  | IADL help from friends/neighbours | | | | | | | | |
| Year (ref.: 2019) | |  |  |  |  |  |  |  |  |
| 2020 | 0.340*** | 0.166*** | 0.101** | 0.310*** | 0.127** | 0.090*** | 0.041 | 0.176*** | 0.099* |
|  | (0.043) | (0.033) | (0.049) | (0.045) | (0.061) | (0.028) | (0.039) | (0.024) | (0.054) |
| 2021 | 0.135*** | 0.089*** | 0.082 | 0.050 | 0.103* | 0.015 | -0.017 | 0.015 | -0.105** |
|  | (0.041) | (0.027) | (0.056) | (0.036) | (0.056) | (0.024) | (0.039) | (0.021) | (0.046) |
| 2022 | -0.010 | -0.001 | -0.026 | -0.034 | -0.028 | -0.034 | -0.015 | 0.004 | -0.109* |
|  | (0.031) | (0.027) | (0.039) | (0.028) | (0.051) | (0.022) | (0.033) | (0.019) | (0.058) |
| # observations | 1,028 | 1,558 | 532 | 850 | 204 | 1,618 | 902 | 2,440 | 538 |
|  | ADL help from children | | | | | | | | |
| Year (ref.: 2019) | |  |  |  |  |  |  |  |  |
| 2021 | 0.017 | 0.046*** | 0.094** | -0.004 | 0.040 | -0.022 | 0.025* | -0.014 | 0.016 |
|  | (0.029) | (0.013) | (0.043) | (0.025) | (0.081) | (0.014) | (0.013) | (0.013) | (0.012) |
| 2022 | 0.023 | 0.008 | 0.042 | -0.023 | 0.024 | 0.032** | 0.011 | -0.013 | 0.004 |
|  | (0.021) | (0.008) | (0.032) | (0.016) | (0.052) | (0.015) | (0.007) | (0.012) | (0.012) |
| # observations | 785 | 1,164 | 417 | 681 | 158 | 1,267 | 697 | 1,822 | 402 |
|  | ADL help from other relatives | | | | | | | | |
| Year (ref.: 2019) | |  |  |  |  |  |  |  |  |
| 2021 | 0.007 | 0.008 | 0.031* | -0.008 | -0.038 | -0.016 | 0.019 | -0.022** | -0.022 |
|  | (0.013) | (0.006) | (0.018) | (0.008) | (0.031) | (0.010) | (0.013) | (0.010) | (0.018) |
| 2022 | 0.017 | -0.009** | -0.002 | -0.010 | -0.026 | 0.018 | 0.002 | -0.008 | -0.017 |
|  | (0.013) | (0.004) | (0.017) | (0.008) | (0.021) | (0.014) | (0.003) | (0.010) | (0.015) |
| # observations | 783 | 1,178 | 416 | 682 | 159 | 1,266 | 696 | 1,829 | 402 |
|  | ADL help from friends/neighbours | | | | | | | | |
| Year (ref.: 2019) | |  |  |  |  |  |  |  |  |
| 2021 | 0.002 | 0.001 | 0.031 | 0.020 | n.a. | 0.005 | 0.005 | 0.015 | 0.006 |
|  | (0.017) | (0.009) | (0.025) | (0.015) |  | (0.006) | (0.010) | (0.015) | (0.031) |
| 2022 | -0.015 | 0.022* | -0.010 | 0.003 | n.a. | 0.005 | -0.003 | 0.006 | -0.029 |
|  | (0.010) | (0.013) | (0.016) | (0.009) |  | (0.008) | (0.007) | (0.007) | (0.021) |
| # observations | 787 | 1,186 | 417 | 682 | n.a. | 1,266 | 700 | 1,837 | 402 |

Notes: *p<0.1, **p<0.05, ***p<0.01. Standard errors in parentheses robust to heteroskedasticity and individual clustering. Results based on Model 2. n.a.: not enough observations to fit the model.

# Appendix 14. Results by country (2/3)

|  | France | Germany | Greece | Hungary | Israel | Italy | Latvia | Lithuania | Luxembourg |  |
| --- | --- | --- | --- | --- | --- | --- | --- | --- | --- | --- |
|  | IADL help from children | | | | | | | | | |
| Year (ref.: 2019) | |  |  |  |  |  |  |  |  |  |
| 2020 | 0.160*** | 0.133*** | 0.244*** | 0.251*** | 0.243*** | 0.281*** | 0.106* | 0.270*** | 0.127* |  |
|  | (0.031) | (0.028) | (0.030) | (0.072) | (0.065) | (0.043) | (0.056) | (0.043) | (0.064) |  |
| 2021 | 0.132*** | 0.138*** | 0.265*** | 0.261*** | 0.280*** | 0.397*** | 0.406*** | 0.266*** | 0.175** |  |
|  | (0.030) | (0.029) | (0.032) | (0.067) | (0.067) | (0.044) | (0.062) | (0.047) | (0.069) |  |
| 2022 | -0.026 | 0.004 | 0.074*** | -0.182*** | 0.017 | 0.013 | -0.002 | 0.002 | -0.079 |  |
|  | (0.022) | (0.021) | (0.028) | (0.046) | (0.047) | (0.038) | (0.052) | (0.029) | (0.053) |  |
| # observations | 1,482 | 1,691 | 1,816 | 567 | 664 | 1,001 | 589 | 878 | 313 |  |
|  | IADL help from other relatives | | | | | | | | | |
| Year (ref.: 2019) | |  |  |  |  |  |  |  |  |  |
| 2020 | 0.142*** | 0.121*** | 0.234*** | 0.293*** | 0.158*** | 0.298*** | 0.077 | 0.172*** | 0.134** |  |
|  | (0.030) | (0.027) | (0.024) | (0.061) | (0.054) | (0.044) | (0.047) | (0.036) | (0.055) |  |
| 2021 | 0.050** | 0.002 | 0.071*** | 0.115** | -0.017 | 0.086** | 0.046 | 0.032 | 0.061 |  |
|  | (0.025) | (0.022) | (0.023) | (0.055) | (0.031) | (0.038) | (0.034) | (0.034) | (0.050) |  |
| 2022 | -0.001 | -0.013 | 0.057*** | 0.006 | 0.083** | 0.034 | 0.017 | 0.009 | -0.046 |  |
|  | (0.018) | (0.022) | (0.022) | (0.031) | (0.039) | (0.032) | (0.032) | (0.024) | (0.038) |  |
| # observations | 1,482 | 1,689 | 1,813 | 565 | 662 | 1,000 | 588 | 872 | 313 |  |
|  | IADL help from friends/neighbours | | | | | | | | | |
| Year (ref.: 2019) | |  |  |  |  |  |  |  |  |  |
| 2020 | 0.145*** | 0.211*** | 0.316*** | 0.478*** | 0.221*** | 0.300*** | 0.137*** | 0.247*** | 0.125* |  |
|  | (0.030) | (0.029) | (0.023) | (0.071) | (0.049) | (0.043) | (0.044) | (0.035) | (0.064) |  |
| 2021 | 0.085*** | 0.120*** | 0.095*** | 0.168** | 0.064* | 0.052 | 0.111** | 0.087*** | 0.083 |  |
|  | (0.029) | (0.031) | (0.021) | (0.079) | (0.037) | (0.037) | (0.048) | (0.029) | (0.054) |  |
| 2022 | -0.029 | 0.032 | 0.020 | -0.047 | -0.013 | -0.009 | 0.055 | 0.010 | -0.005 |  |
|  | (0.023) | (0.021) | (0.016) | (0.040) | (0.022) | (0.034) | (0.038) | (0.022) | (0.050) |  |
| # observations | 1,482 | 1,689 | 1,813 | 564 | 662 | 998 | 589 | 873 | 313 |  |
|  | ADL help from children | | | | | | | | | |
| Year (ref.: 2019) | |  |  |  |  |  |  |  |  |  |
| 2021 | 0.000 | 0.022 | 0.095*** | 0.037 | -0.013 | 0.081** | 0.122*** | 0.099*** | 0.060** |  |
|  | (0.010) | (0.017) | (0.024) | (0.066) | (0.044) | (0.033) | (0.042) | (0.032) | (0.027) |  |
| 2022 | -0.007 | 0.015 | 0.012 | -0.031 | 0.028 | 0.004 | 0.013 | 0.001 | 0.010 |  |
|  | (0.015) | (0.015) | (0.019) | (0.023) | (0.032) | (0.029) | (0.019) | (0.018) | (0.021) |  |
| # observations | 1,161 | 1,301 | 1,338 | 469 | 535 | 747 | 475 | 664 | 228 |  |
|  | ADL help from other relatives | | | | | | | | | |
| Year (ref.: 2019) | |  |  |  |  |  |  |  |  |  |
| 2021 | 0.001 | 0.015 | 0.020 | -0.026 | 0.014 | -0.009 | 0.052 | 0.034** | -0.018 |  |
|  | (0.006) | (0.013) | (0.013) | (0.029) | (0.016) | (0.012) | (0.035) | (0.016) | (0.022) |  |
| 2022 | 0.004 | -0.006 | -0.004 | -0.046 | 0.015 | 0.003 | -0.012 | 0.015 | -0.031 |  |
|  | (0.008) | (0.007) | (0.010) | (0.028) | (0.015) | (0.011) | (0.019) | (0.011) | (0.021) |  |
| # observations | 1,169 | 1,307 | 1,347 | 472 | 532 | 751 | 471 | 664 | 230 |  |
|  | ADL help from friends/neighbours | | | | | | | | | |
| Year (ref.: 2019) | |  |  |  |  |  |  |  |  |  |
| 2021 | 0.006 | 0.039** | 0.042*** | 0.026 | n.a. | 0.025** | 0.108*** | 0.024 | 0.005 |  |
|  | (0.005) | (0.017) | (0.013) | (0.021) |  | (0.012) | (0.039) | (0.016) | (0.034) |  |
| 2022 | 0.003 | -0.004 | 0.003 | -0.010 | n.a. | 0.003 | 0.004 | 0.005 | -0.021 |  |
|  | (0.003) | (0.006) | (0.006) | (0.011) |  | (0.011) | (0.014) | (0.008) | (0.016) |  |
| # observations | 1,174 | 1,308 | 1,349 | 473 | n.a. | 751 | 476 | 664 | 230 |  |

Notes: *p<0.1, **p<0.05, ***p<0.01. Standard errors in parentheses robust to heteroskedasticity and individual clustering. Results based on Model 2. n.a.: not enough observations to fit the model.

# Appendix 15. Results by country (3/3)

|  | Malta | Netherlands | Poland | Romania | Slovakia | Slovenia | Spain | Sweden | Switzerland |
| --- | --- | --- | --- | --- | --- | --- | --- | --- | --- |
|  | IADL help from children | | | | | | | | |
| Year (ref.: 2019) | |  |  |  |  |  |  |  |  |
| 2020 | 0.203** | 0.047 | 0.161*** | 0.118** | 0.244*** | 0.078** | 0.239*** | 0.323*** | 0.361*** |
|  | (0.087) | (0.050) | (0.038) | (0.053) | (0.079) | (0.034) | (0.040) | (0.048) | (0.041) |
| 2021 | 0.127* | 0.047 | 0.295*** | 0.240*** | 0.492*** | 0.163*** | 0.427*** | 0.351*** | 0.258*** |
|  | (0.074) | (0.056) | (0.041) | (0.057) | (0.083) | (0.034) | (0.054) | (0.056) | (0.044) |
| 2022 | -0.078 | 0.096*** | 0.027 | 0.047 | 0.024 | -0.012 | 0.008 | 0.116*** | 0.007 |
|  | (0.065) | (0.036) | (0.025) | (0.049) | (0.056) | (0.027) | (0.038) | (0.036) | (0.039) |
| # observations | 195 | 654 | 1,320 | 713 | 385 | 1,485 | 962 | 970 | 809 |
|  | IADL help from other relatives | | | | | | | | |
| Year (ref.: 2019) | |  |  |  |  |  |  |  |  |
| 2020 | 0.077 | -0.046 | 0.148*** | 0.018 | 0.274*** | 0.040 | 0.143*** | 0.160*** | 0.206*** |
|  | (0.055) | (0.043) | (0.036) | (0.040) | (0.077) | (0.032) | (0.038) | (0.040) | (0.042) |
| 2021 | 0.102 | -0.076* | 0.042 | 0.064 | 0.077 | -0.042 | 0.166*** | 0.007 | 0.016 |
|  | (0.095) | (0.045) | (0.027) | (0.043) | (0.069) | (0.027) | (0.042) | (0.038) | (0.030) |
| 2022 | 0.050 | -0.029 | 0.005 | -0.013 | -0.037 | -0.013 | -0.058** | 0.018 | -0.069** |
|  | (0.084) | (0.029) | (0.021) | (0.029) | (0.051) | (0.025) | (0.026) | (0.027) | (0.028) |
| # observations | 195 | 654 | 1,318 | 713 | 385 | 1,483 | 960 | 969 | 807 |
|  | IADL help from friends/neighbours | | | | | | | | |
| Year (ref.: 2019) | |  |  |  |  |  |  |  |  |
| 2020 | 0.155*** | 0.002 | 0.182*** | 0.107*** | 0.475*** | 0.141*** | 0.187*** | 0.294*** | 0.347*** |
|  | (0.052) | (0.052) | (0.034) | (0.040) | (0.067) | (0.025) | (0.034) | (0.049) | (0.045) |
| 2021 | 0.008 | 0.004 | 0.078*** | 0.017 | 0.225*** | 0.053** | 0.152*** | 0.125** | 0.245*** |
|  | (0.063) | (0.043) | (0.027) | (0.047) | (0.065) | (0.026) | (0.039) | (0.053) | (0.041) |
| 2022 | 0.023 | -0.018 | 0.014 | -0.003 | 0.050 | 0.037 | 0.004 | -0.005 | -0.009 |
|  | (0.044) | (0.030) | (0.017) | (0.027) | (0.050) | (0.025) | (0.020) | (0.042) | (0.032) |
| # observations | 195 | 654 | 1,316 | 713 | 386 | 1,482 | 960 | 969 | 807 |
|  | ADL help from children | | | | | | | | |
| Year (ref.: 2019) | |  |  |  |  |  |  |  |  |
| 2021 | -0.070 | 0.073** | 0.070*** | -0.023 | 0.174*** | -0.019 | 0.104*** | 0.033 | 0.021* |
|  | (0.043) | (0.036) | (0.025) | (0.035) | (0.065) | (0.017) | (0.031) | (0.026) | (0.012) |
| 2022 | -0.043 | 0.001 | -0.003 | -0.032 | -0.046 | -0.005 | 0.036 | 0.014* | 0.005 |
|  | (0.032) | (0.013) | (0.014) | (0.029) | (0.049) | (0.015) | (0.029) | (0.008) | (0.009) |
| # observations | 146 | 572 | 1,076 | 530 | 299 | 1,142 | 746 | 787 | 610 |
|  | ADL help from other relatives | | | | | | | | |
| Year (ref.: 2019) | |  |  |  |  |  |  |  |  |
| 2021 | 0.019 | -0.034 | 0.023 | 0.004 | -0.007 | -0.009 | 0.047 | n.a. | -0.007 |
|  | (0.021) | (0.029) | (0.017) | (0.023) | (0.024) | (0.013) | (0.032) |  | (0.006) |
| 2022 | 0.011 | -0.028 | -0.014* | 0.008 | -0.028 | -0.001 | -0.021 | n.a. | 0.011 |
|  | (0.015) | (0.021) | (0.008) | (0.017) | (0.028) | (0.011) | (0.017) |  | (0.010) |
| # observations | 146 | 573 | 1,079 | 534 | 299 | 1,142 | 751 | n.a. | 614 |
|  | ADL help from friends/neighbours | | | | | | | | |
| Year (ref.: 2019) | |  |  |  |  |  |  |  |  |
| 2021 | n.a. | 0.034* | 0.011 | 0.030 | 0.024 | 0.005 | 0.003 | 0.027 | 0.023* |
|  |  | (0.019) | (0.008) | (0.026) | (0.028) | (0.011) | (0.003) | (0.019) | (0.012) |
| 2022 | n.a. | -0.005 | -0.005 | 0.013 | -0.041 | -0.000 | 0.004 | 0.000 | 0.003 |
|  |  | (0.005) | (0.004) | (0.014) | (0.029) | (0.007) | (0.004) | (0.004) | (0.008) |
| # observations | n.a. | 578 | 1,079 | 534 | 300 | 1,143 | 751 | 792 | 617 |

Notes: *p<0.1, **p<0.05, ***p<0.01. Standard errors in parentheses robust to heteroskedasticity and individual clustering. Results based on Model 2. n.a.: not enough observations to fit the model.
